# Linking Signal Relevancy and Intensity in Predictive Tactile Suppression

**DOI:** 10.3389/fnhum.2022.795886

**Published:** 2022-02-24

**Authors:** Marie C. Beyvers, Lindsey E. Fraser, Katja Fiehler

**Affiliations:** ^1^Department of Psychology, Justus Liebig University Giessen, Giessen, Germany; ^2^Center for Vision Research, York University, Toronto, ON, Canada; ^3^Department of Psychology, York University, Toronto, ON, Canada; ^4^Center for Mind, Brain and Behavior, University of Marburg and Justus Liebig University Giessen, Giessen, Germany

**Keywords:** tactile suppression, prediction, feedback, goal-directed movement, task-relevance

## Abstract

Predictable somatosensory feedback leads to a reduction in tactile sensitivity. This phenomenon, called *tactile suppression*, relies on a mechanism that uses an efference copy of motor commands to help select relevant aspects of incoming sensory signals. We investigated whether tactile suppression is modulated by (a) the task-relevancy of the predicted consequences of movement and (b) the intensity of related somatosensory feedback signals. Participants reached to a target region in the air in front of a screen; visual or tactile feedback indicated the reach was successful. Furthermore, tactile feedback intensity (strong vs. weak) varied across two groups of participants. We measured tactile suppression by comparing detection thresholds for a probing vibration applied to the finger either early or late during reach and at rest. As expected, we found an overall decrease in late-reach suppression, as no touch was involved at the end of the reach. We observed an increase in the degree of tactile suppression when strong tactile feedback was given at the end of the reach, compared to when weak tactile feedback or visual feedback was given. Our results suggest that the extent of tactile suppression can be adapted to different demands of somatosensory processing. Downregulation of this mechanism is invoked only when the consequences of missing a weak movement sequence are severe for the task. The decisive factor for the presence of tactile suppression seems not to be the predicted action effect as such, but the need to detect and process anticipated feedback signals occurring during movement.

## Introduction

Somatosensory feedback plays a crucial role in the control of goal-directed movements. However, delays and noise associated with processing of afferent signals constantly arising from the moving body part can compromise the detectability of somatosensory feedback (Miall and Wolpert, 1996; Faisal et al., 2008). Selecting relevant aspects of incoming sensory information is fundamental for efficient signal processing. It has been suggested that, during movement, somatosensory feedback is combined with predictions about future sensory states of the moving body part in order to compensate for ongoing sensorimotor noise (Desmurget and Grafton, 2000). These predictions are formed on the basis of an efference copy of motor commands in a feedforward fashion (Wolpert and Flanagan, 2001). When somatosensory feedback can be precisely predicted, reliance on such feedback can be minimized, resulting in a decrease in tactile sensitivity (Williams and Chapman, 2002; Bays et al., 2006; Chapman and Beauchamp, 2006). This mechanism, also referred to as *tactile suppression*, has been investigated during self-touch (Blakemore et al., 1998; Bays et al., 2005; Walsh et al., 2011; Kilteni and Ehrsson, 2017), showing that self-generated stimuli are perceived as less intense compared to externally generated stimuli. Externally generated stimuli are also harder to detect during movement, compared to a resting state (Chapman et al., 1987; Fraser and Fiehler, 2018; Gertz et al., 2018; Manzone et al., 2018; Voudouris and Fiehler, 2021). Importantly, this relative suppression is stronger for predicted compared to unpredicted somatosensory consequences of movement (Voudouris et al., 2019; Führer et al., 2021). In sum, tactile suppression provides a well-established behavioral measure of the strength of somatosensory predictions and allows us to understand how the brain utilizes predictions of future sensory states to efficiently process signals resulting from goal-directed movements.

Tactile suppression can be modulated by various factors. Some studies have reported a positive correlation between movement speed and the magnitude of tactile suppression. For example, Cybulska-Klosowicz and colleagues tested detection of a mild electrical stimulus applied to the finger during elbow extension movements of varying speeds (Cybulska-Klosowicz et al., 2011). They found no evidence of tactile suppression at very slow movement speeds (< 50 mm/s), whereas at higher speeds detection thresholds increased, indicating substantive tactile suppression. Moreover, tactile suppression decreases during haptic exploration (Juravle et al., 2013), which is typically associated with slower movement speeds. Other studies measuring the time course of suppression in goal-directed movements have reported an increase in tactile suppression early in the course of a movement, when movement speed is typically higher (Juravle et al., 2010; Colino and Binsted, 2016). These results are in conflict with more recent findings showing a release from suppression at the time of peak speed (Voudouris and Fiehler, 2021). Thus, it is still unclear how exactly movement speed can modulate tactile suppression or whether there are other factors influencing this relationship.

In a previous study, we sought to examine this ambiguity in more detail by determining whether implicitly generated differences in movement speed had a direct effect on tactile suppression (Fraser and Fiehler, 2018). Participants performed speeded reaching movements to a visual target. By manipulating the target’s size, we systematically varied reach kinematics, capitalizing on Fitts’ law (Fitts, 1954; Mackenzie et al., 1987), without changing the reach goal or explicit instructions between conditions. We measured suppression of a vibrotactile stimulus applied either early in reach (25% reach time), when higher speeds are expected, or late (75% reach time), when lower speeds are expected. In all conditions, participants significantly slowed down toward the end of the reach (by varying amounts, depending on target size). Despite this, we found an increase in the magnitude of tactile suppression at the later stages of movement, independent of target size. These results support other studies also showing stronger suppression at the end of goal-directed movements (Juravle et al., 2018; Voudouris and Fiehler, 2021). In a follow-up experiment (Fraser and Fiehler, 2018), we tested whether the expectation of a tactile consequence at the end of the reach would further increase late-reach tactile suppression. Participants reached to visual targets of different colors associated with or without a tactile consequence. We found a selective increase in late-reach suppression only when a tactile consequence of movement was expected. We concluded that tactile suppression is modulated by the expected tactile consequences of movement in a time-sensitive manner. In other words, the ability to detect tactile stimuli seems to depend on a dynamic modulation of suppression, based on the predictability of anticipated feedback signals.

The previously described experiment, along with work showing certain predicted consequences of movement (e.g., expected force; Broda et al., 2020) do not likely modulate tactile suppression, raises questions about the exact nature of dynamic modulation of suppression. Predictability does not appear to be the sole factor modulating the strength of tactile suppression. Instead, specific predictable features of a movement seem to have more influence over modulation of suppression than others. One such influential feature is the *task-relevance* of a predicted sensory outcome. In our previous study, the tactile feedback which led to increased suppression was highly redundant. Visual cues indicating the successful end of the reach were always present. Thus, the selective increase in late-reach suppression we observed might be attributed to a lack of task-relevance of this tactile feedback. There is evidence that the strength of tactile suppression is indeed diminished in contexts where one is required to process somatosensory feedback signals, specifically at task-relevant effectors. For instance, during grasping, the magnitude of suppression is lowest in the fingers involved in the grasp (Colino and Binsted, 2016; Manzone et al., 2018). Furthermore, suppression is diminished during haptic exploration, when tactile information needs to be actively sampled (Juravle et al., 2013). It appears that the modulation of tactile suppression includes an adjustment for signals that, although highly predictable, remain informative and therefore useful.

In addition, the *intensity* of somatosensory feedback may have a potential influence on the dynamic modulation of tactile suppression. When uncertainty about somatosensory input occurs, predictive processes are downregulated to improve the processing of incoming sensory signals (Franklin et al., 2012). Decreasing the intensity of tactile feedback may increase the upweighting of input signals to avoid missing the sensory feedback. Such a strategy would necessarily be accompanied by a reduction in tactile suppression.

In the present study, we investigated whether tactile sensitivity during goal-directed movements is modulated by both the task-relevancy of the predicted consequences of movement and the intensity of related feedback signals. First, to increase task-relevancy of the tactile feedback, we employed a novel task in which (a) given feedback was uniquely informative about task completion, and (b) additional visual information about the target location was not provided during the movement. Second, we eliminated all other expected tactile signals at the end of the reach as a possible confound by instructing participants to reach close to the screen without touching it. We kept feedback uniquely informative by having visual or tactile feedback as the only indication that a target was found. Thus, a predictable tactile component at the end of the movement was only present with tactile feedback. Visual feedback served as a control to highlight the influence of a predictable, yet task-relevant tactile feedback on the strength of somatosensory predictions. Additionally, we examined further modulation of tactile suppression by varying the intensity of the tactile feedback (strong vs. weak) across two separate groups of participants.

The ability to detect tactile signals was assessed early in the movement, when no feedback was predicted (∼25% reaching time) and late in the movement, when anticipated feedback became more likely (∼75% reaching time). As participants had to control and stop their movements before touching the screen, we expected an overall reduction in late-reach suppression for both conditions. We further hypothesized a relative increase in suppression in conditions where predictable tactile feedback occurred at the end of the movement, compared to when feedback was visual. We expected this increase in suppression to be most profound for the strong tactile feedback, which should result in better predictions and therefore more suppression of incoming signals. These results would indicate that both the need to process somatosensory information, and the anticipated intensity of this information, modulates suppression of predicted somatosensory signals.

## Materials and Methods

### Participants

A total of 68 students participated in the study and completed the experiment. The sample size was not calculated *a priori*, but was chosen to be comparable to previous studies of tactile suppression (e.g., Fraser and Fiehler, 2018; Gertz et al., 2018; Manzone et al., 2018; Voudouris et al., 2019; Führer et al., 2021). In exchange for their participation, participants received either course credit, or financial compensation at the rate of eight Euro/hour. Owing to exclusion criteria (see section “Data Analysis”), the final sample consisted of 52 participants divided into two groups that received different intensities of tactile feedback during the experiment [strong tactile feedback: *n* = 26 (18 f, 8 m), age = 23 ± 3; weak tactile feedback: *n* = 26 (17 f, 9 m), age = 23 ± 3]. Participants were all right-handed, as assessed by the German translation of the Edinburgh Handedness Inventory (Oldfield, 1971; strong tactile feedback: 95, weak tactile feedback: 96). The experiment was approved by the research ethics board at Justus Liebig University Giessen, and was run in accordance with the Declaration of Helsinki (2008).

### Apparatus

**FIGURE 1:**
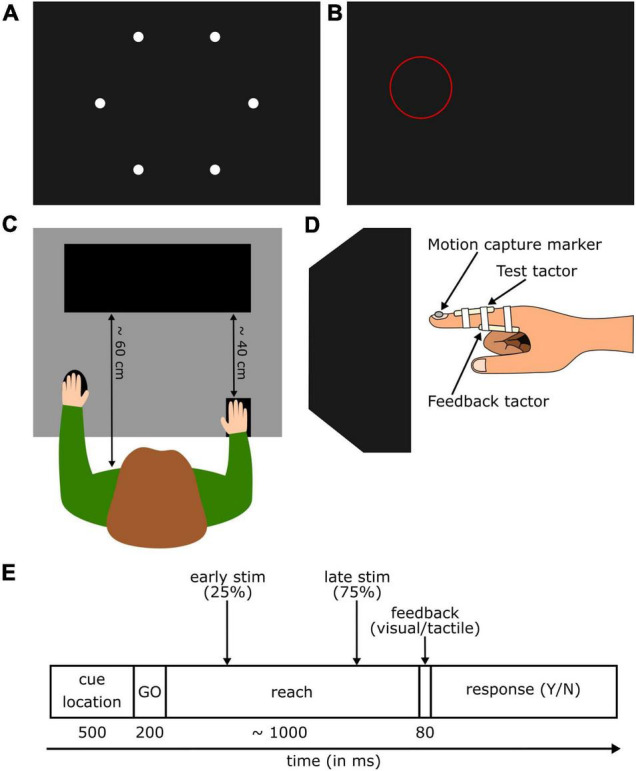
**(A)** Display of all possible target locations on the screen. **(B)** Example for a location cue (red circle) presented at the beginning of each trial. The cue was positioned so that the target was always in its center. The target was never visible during the experiment. **(C)** Top view of the setup showing a participant sitting in front of the screen with the right hand on the start position and the left hand on the response buttons. **(D)** Side view of the right index finger hovering in front of the screen. **(E)** Time course of a trial in the detection block. After the presentation of the cue location and a go cue, participants reached to the target with their right hand. A short vibrotactile probing stimulus was presented either early or late during the movement. After finding the target and receiving the feedback, participants indicated whether they felt the probing stimulus or not.

Participants were seated at an 80 × 117 cm table (Figure 1C) approximately 60 cm in front of the monitor (ViewPixx/3D, VPixx Technologies Inc., Saint-Bruno, Canada). A small keypad (12.5 × 8 cm) was placed at the edge of the table closest to the participant, under their right hand (approximately 40 cm from the screen). A wireless mouse was fixed to the table at the same distance, and participants placed their left hand on it throughout the experiment. The participant’s right hand was fitted with two custom-built vibrotactile stimulators (“tactors”; Engineer Acoustics Inc., Florida, United States), each with a 5 mm diameter vibration pad. The first tactor was attached to the dorsal surface of the right index finger, such that the pad rested on the skin, roughly equidistant between the proximal and distal interphalangeal joints (test tactor). The second tactor was attached to the ventral surface of the participant’s finger just distal of the metacarpophalangeal joint (feedback tactor). The tactors were controlled using a custom MATLAB (Mathworks, Natick, United States) toolbox developed by Engineering Acoustics Inc. A small infrared-emitting diode was attached to the participant’s finger; an Optotrak Certus (Northern Digital, Waterloo, Ontario, CA) mounted on the wall tracked the motion of the diode at a sampling rate of 100 Hz. Motion tracking was controlled via MATLAB using the MOTOM toolbox (Derzsi and Volcic, 2018). Tactors and wires were held in place with medical adhesive tape (Figure 1D).

### Procedure

Before starting the experiment, participants completed a *calibration* block. Six white circles (0.5 cm radius) distributed in a ring around the center of the screen (Figure 1A) were presented one after the other. The first circle’s location was determined for each participant by a random number generator, and the following five circles were equally spaced apart. When a circle appeared, participants were instructed to hold their finger in the air in front of the circle without touching the screen (Figure 1D). Once their hand was in position, they pressed a mouse button and the recorded finger location generated an invisible “target” location in the air. After recording all six target locations, a test phase began in which participants again had to reach to the targets in a randomized order. When participants felt their finger was in the right location, they clicked the mouse, and the fingertip location was recorded again. The test was considered successful if they recreated their previous pointing location with a 15 mm margin of error in every direction. If all six locations did not pass the testing, the calibration was repeated.

The experiment started with a *baseline* block, where participants kept their right ring finger pressed down a button on the keypad (start button). A ring (4 cm radius, line width 0.015 cm) flashed on the screen for 500 ms around one of the six target locations (location cue, Figure 1B); the target itself was never visible. Immediately after that two consecutive tones (100 ms, 1,000 Hz) were presented separated by a 2 s delay. In the middle of the delay, the test tactor vibrated for a 50 ms pulse (vibration trials) or kept silent (no-vibration trials). Participants were instructed to keep their hand still throughout the trial. Following the second tone, an onscreen message prompted participants to indicate if they had felt a vibration from the test tactor, using the mouse to respond yes or no. Five vibration intensities were tested (peak-to-peak displacement of 1.66, 4.98, 8.3, 11.63, and 14.96 μm), each delivered five times in a random order. In addition, five no-vibration catch trials were randomly interspersed throughout the test, leading to 30 trials in total.

Figure 1E illustrates the trial progression. Participants completed two *detection* task blocks in a pseudorandomly assigned order. At the start of a trial, participants waited with their middle and ring finger on the start button. A location cue flashed for 500 ms at on one of the six target locations; location cue characteristics were the same as in the baseline block. After the location cue disappeared, a go cue sounded for 200 ms and instructed participants to reach out with their right index finger to find the invisible target. Hand trajectory was recorded using the Optotrak. When the participant’s fingertip reached the target, feedback was given. In the *tactile feedback* block, the feedback tactor on the base of the finger would emit an 80 ms pulse. One group of participants received strong tactile feedback (peak-to-peak amplitude of 134.7 μm, approximately 1,007% of the detection threshold during movement). This value was chosen to be very intense and thus clearly noticeable. The other group received weak tactile feedback (peak-to-peak amplitude of 18.29 μm, approximately 152% of the detection threshold during movement). In a pilot experiment this value was identified as perceptually “faint” yet still detectable. In both cases the feedback vibration was the only indication that the target was found. In the *visual feedback* block, a message (“Target found”) appeared in the center of the screen when the target was found. After receiving the feedback, participants returned to the keypad and pressed the start button again. The screen prompted them to respond whether they felt a vibration on the top of their right index finger during the movement. Participants responded using the mouse to indicate yes or no. Upon response, the next trial began.

Each detection block started with five practice trials wherein no stimulation was delivered. These were used to generate initial stimulation times for the first test trials. Following practice, trials could contain a 50 ms vibration from the test tactor at one of two time points. *Stimulation time* was calculated using either the 25% (early stimulation) or 75% (late stimulation) of the median of the previous five reaches. Eight vibration intensities (peak-to-peak displacement of 1.66, 4.98, 8.3, 11.63, 14.96, 18.29, 21.62, and 24.95 μm) were tested five times, in random order, at each of the two time points during movement. In addition, 16 no-stimulation catch trials were randomly interspersed in the trial order, leading to 96 initial trials for each block. In each trial, participants had 3 s to find the target location. If they took more than 3 s, a message (“Too slow”) appeared on the screen. In this case, or if participants reached too quickly and the test stimulus did not fire, or if the stimulus fired during the last 150 ms of the movement, the trial was discarded and added to the end of the block. If participants reached too quickly and received an early stimulus in the second half of the reach time, the trial was reassigned to the late condition and a subsequent late trial of the same vibration intensity was removed. The early trial was added to the end of the block. If participants reached too slowly and received a late stimulus in the first half of the reach time, the trial was reassigned to the early condition, and a subsequent early trial with the same vibration intensity was removed from the block. The late trial was then repeated at the end of the block.

### Data Analysis

Data preprocessing was conducted with MATLAB R2019b (MathWorks, Inc., Natick, MA). Motion capture data for a given trial included x, y and z coordinates of the marker from the right index finger from the sounding of the go cue until the target was found. These three vectors were dual-pass filtered using the MATLAB filtfilt function using a 2nd order lowpass butterworth filter with a cutoff of.30. The speed of the reaching index finger over the course of the reaching movement was calculated by numerical differentiation of x, y and z velocity. For each participant, speed data were averaged for each feedback type x stimulation time condition, resulting in four reach speed profiles (tactile-early, tactile-late, visual-early and visual-late). *Speed at stimulation* was extracted as the speed of the index finger at the timepoint when the test stimulus was triggered. Speed at stimulation was subjected to a repeated measures ANOVA comparing feedback type (visual, tactile), stimulation time (early, late), and tactile feedback intensity (strong, weak) to determine whether any of these factors influenced movement speed at critical moments in the task. *Reach time* was calculated as the time between the go cue was given and the participant found the target. Reach time was assessed using a repeated measures ANOVA comparing feedback type (visual, tactile), stimulation time (early, late) and tactile feedback intensity (strong, weak) to determine whether any of these factors influenced the length of time a trial took to complete.

To analyze perceptual behavior, we fitted the proportion of trials in which the probing stimulus was perceived for the baseline and each experimental condition (feedback type x stimulation time x tactile feedback intensity) to a logistic function using maximum-likelihood estimation with the function psignifit (Wichmann and Hill, 2001) in MATLAB. The 50% detection threshold (point of subjective equality, PSE), which refers to the threshold of detectability, as well as the difference between the 50 and the 84% threshold (just noticeable difference, JND), which corresponds to one SD from the PSE and refers to the precision of the judgment, were extracted for each condition. Suppression scores were generated by subtracting the baseline PSE from the PSE of each movement condition. As with suppression, precision scores were formed by differences of the corresponding JNDs. Higher positive values for both suppression and precision scores indicated stronger tactile suppression. Participants showing a false alarm rate of 30% or higher (*n* = 12) or a baseline detection threshold or suppression scores that were ± 2.5 standard deviations from the group mean (n = 4) were excluded from all analyses. Tactile suppression scores (*n* = 52) were first compared in a one-tailed *t*-test, to determine whether significant tactile suppression occurred in each condition. Furthermore, suppression and precision scores were each subjected to a repeated measures ANOVA with feedback type (visual, tactile), stimulation time (early, late), and tactile feedback intensity (strong, weak) to determine whether any of these factors influenced the detection of the probing stimuli. Finally, to control for a relation of speed (Cybulska-Klosowicz et al., 2011; Fraser and Fiehler, 2018) and the amount of tactile suppression we correlated participants’ reaching speed at each stimulation time, with their suppression score for that time. This was performed separately for each feedback type x stimulation time x tactile feedback intensity condition.

All statistical analyses were carried out with JASP (Version 0.14.1). Significant interactions were inspected with *post-hoc t*-tests, Bonferroni-corrected for multiple comparisons (α = 0.008). Effect sizes are described as partial Eta squared for ANOVAs and Cohen’s d for *t*-tests.

## Results

**FIGURE 2:**
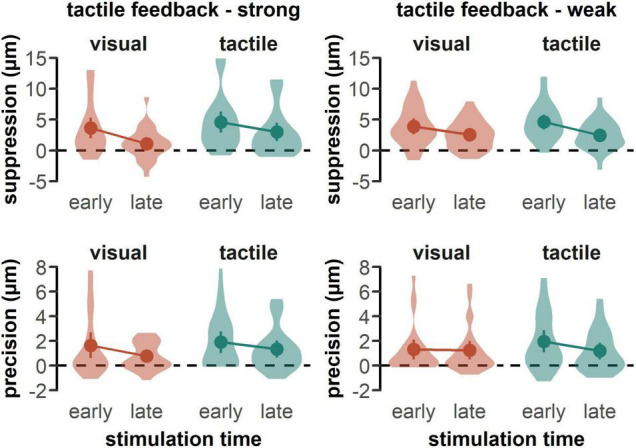
Mean suppression and precision scores by feedback type. Data is plotted separately for both groups receiving a strong tactile feedback **(left panel)** and a weak tactile feedback **(right panel)**. Higher suppression and precision scores indicate a deterioration of tactile perception. Colored dots represent the mean across participants with error bars indicating the standard error of the mean. Shaded areas represent the data distribution smoothed with a kernel density function.

In line with previous studies (Fraser and Fiehler, 2018; Gertz et al., 2018; Manzone et al., 2018; Voudouris and Fiehler, 2021), tactile sensitivity was impeded in all reaching conditions, as all suppression scores were significantly greater than zero for both the group with the strong tactile feedback in the visual-early, *t*(25) = 4.37, *p* < 0.001, *d* = 0.86, visual-late, *t*(25) = 2.12, *p* = 0.004, *d* = 0.42, tactile-early, *t*(25) = 5.39, *p* < 0.001, *d* = 1.06, and tactile-late condition, *t*(25) = 4.01, *p* < 0.001, *d* = 0.79 (see Figure 2, upper left panel), as well as for the group with the weak tactile feedback in the visual-early, *t*(25) = 5.52, *p* < 0.001, *d* = 1.08, visual-late, *t*(25) = 5.55, *p* < 0.001, *d* = 1.09, tactile-early, *t*(25) = 6.99, *p* < 0.001, *d* = 1.37, and tactile-late, *t*(25) = 5.19, *p* < 0.001, *d* = 1.02, condition (see Figure 2, upper right panel). Further, detection precision was significantly greater than zero in all reaching conditions for both the group with the strong tactile feedback in the visual-early, *t*(25) = 2.95, *p* = 0.007, *d* = 0.58, visual-late, *t*(25) = 3.68, *p* = 0.001, *d* = 0.72, tactile-early, *t*(25) = 3.95, *p* < 0.001, *d* = 0.81, and tactile-late condition, *t*(25) = 3.95, *p* < 0.001, *d* = 0.77 (see Figure 2, lower left panel), as well as for the group with the weak tactile feedback in the visual-early, *t*(25) = 3.41, *p* = 0.002, *d* = 0.67, visual-late, *t*(25) = 3.37, *p* = 0.003, *d* = 0.66, tactile-early, *t*(25) = 4.25, *p* < 0.001, *d* = 0.83, and tactile-late, *t*(24) = 3.10, *p* = 0.005, *d* = 0.62, condition (see Figure 2, lower right panel).

**FIGURE 3:**
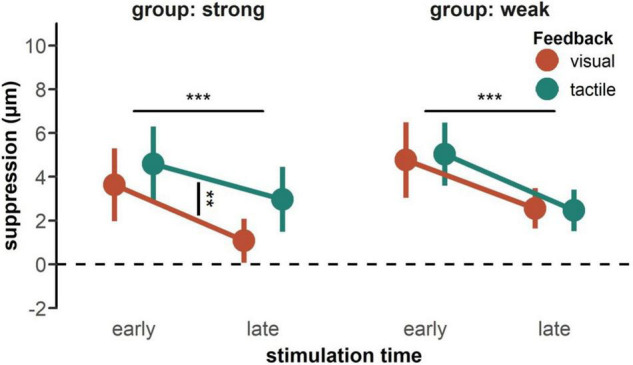
Interaction of suppression scores by feedback type and feedback intensity. Data is plotted separately for both groups receiving a strong tactile feedback **(left panel)** and a weak tactile feedback **(right panel)**. Colored dots represent the mean across participants with error bars indicating the standard error of the mean. Only the group receiving strong tactile feedback showed a difference in suppression between feedback types. Statistics performed by a rm-ANOVA including feedback type (visual, tactile), stimulation time (early, late), and tactile feedback intensity (strong, weak), **p < 0.01, ***p < 0.001.

Participants showed a stronger suppression effect, which represent a decrease in detection threshold relative to baseline, when the feedback was tactile than when it was visual [*F*(1, 50) = 8.27, *p* = 0.006, $\eta_{p}^{2}$ = 0.14] (see Figure 2, upper panels), and when the stimulation occurred in the early than the late phase of the movement [*F*(1, 50) = 35.69, *p* < 0.001, $\eta_{p}^{2}$ = 0.42]. Further, we found a significant interaction between feedback type and tactile feedback intensity [*F*(1, 50) = 5.47, *p* = 0.015, $\eta_{p}^{2}$ = 0.11]. *Post-hoc t*-test revealed a significant difference between visual and tactile feedback only in case of strong tactile feedback, *t*(25) = −3.82, *p*_bonf_ = 0.002, *d* = 0.75 (see Figure 3). When tactile feedback was strong, participants showed more suppression for tactile than for visual feedback (tactile: *M* = 3.78, *SD* = 4.11; visual: *M* = 2.35, *SD* = 3.70), whereas when tactile feedback was weak, there was no difference between feedback conditions (tactile: *M* = 3.75, *SD* = 3.34; visual: *M* = 3.66, *SD* = 3.66). There was no other significant main effect or interaction. In line with the suppression scores, the detection precision thresholds decreased relative to baseline in the late phase, compared to the early phase of the movement [*F*(1, 50) = 8.22, *p* = 0.006, $\eta_{p}^{2}$ = 0.14] (see Figure 2, lower panels). We found no other systematic differences in the precision scores.

**FIGURE 4:**
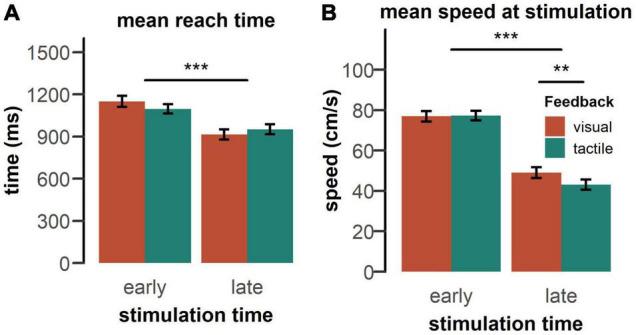
**(A)** Mean reach time and **(B)** mean movement speed at stimulation by feedback type pooled over both groups. Reaches took longer, if a probing stimulus occurred in the early phase of the movement and participants moved faster during the time of early stimulation. Vertical bars indicate standard error of the mean. Statistics performed by a rm-ANOVA including feedback type (visual, tactile), stimulation time (early, late), and tactile feedback intensity (strong, weak), **p < 0.01, ***p < 0.001.

Average reach time and average speed at stimulation are shown in Figure 4. Reach times, reflecting the time from the start of the movement until the target was found, were dependent on the timing of the probing stimulus, [*F*(1, 50) = 113.48, *p* < 0.001, $\eta_{p}^{2}$ = 0.69]. Receiving a probe in the late phase of the movement significantly reduced reaching time, compared to when it was presented in the early phase of the movement. There was also a significant feedback type x stimulation time interaction [*F*(1, 50) = 20.95, *p* < 0.001, $\eta_{p}^{2}$ = 0.30]. The difference in reach time between the early and late stimulation condition was more pronounced with visual feedback, compared to tactile feedback, *t*(51) = 4.60, *p* < 0.001, *d* = 0.64. No other main effect or interaction was significant.

**FIGURE 5:**
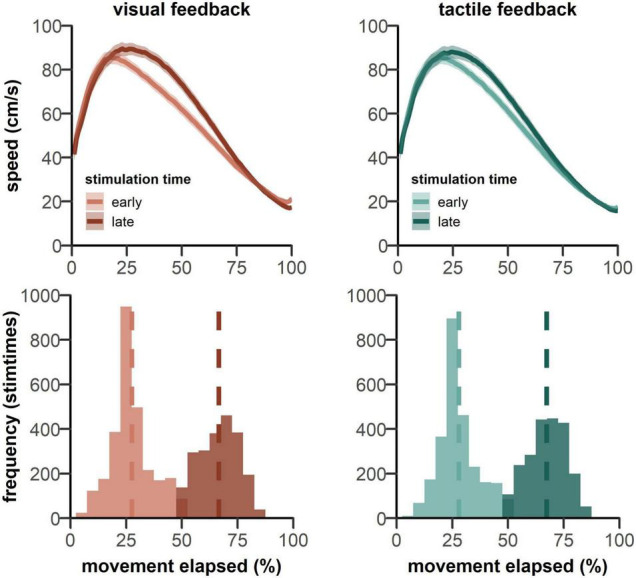
**(top panels)** Show the mean movement speed across trial time, starting from the go signal. Data is plotted separately for both feedback types pooled over both groups. Participants show an earlier reduction in movement speed when the probing stimulus was delivered in the early phase of the movement (lighter colored lines). Shaded patches indicate standard error of the mean. **(lower panels)** Show the frequency of stimulation times over all trials. The dashed lines show the mean stimulation time for each condition.

As expected on the basis of the typical speed profile of a goal-directed movement (see Figure 5), the speed at the moment of receiving the tactile stimulation was substantially higher in the early phase of the movement, compared to the late phase [*F*(1, 50) = 176.60, *p* < 0.001, $\eta_{p}^{2}$ = 0.78] (Figure 4). There was also a significant feedback type x stimulation time interaction [*F*(1,50) = 19.55, *p* < 0.001, $\eta_{p}^{2}$ = 0.28]. For the late stimulation condition, participants moved slower in anticipation of tactile feedback, compared to visual feedback, *t(25)* = 3.52, *p*_bonf_ = 0.005, *d* = 0.40, whereas there was no difference in movement speed in the early stimulation condition. No other main effect or interaction was significant.

For stimulation times, 25 and 75% of movement times were targeted. In fact, across all trials, stimulations occurred on average at 27.48 and 66.58% of the movement for visual feedback and at 27.88 and 67.36% for tactile feedback (see Figure 5). Thus, stimulation times for both feedback conditions were comparable and were in the early and late phases of reaching movement.

**FIGURE 6:**
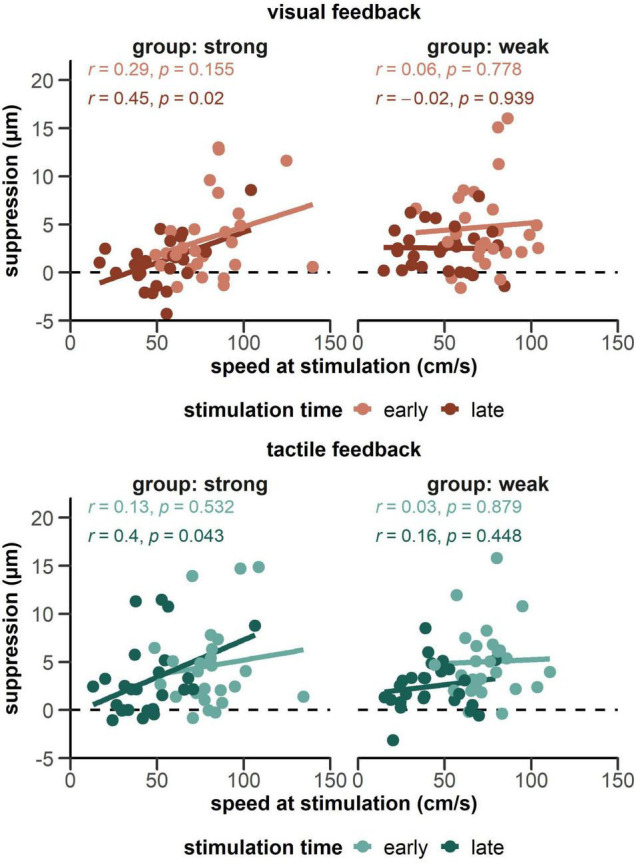
Correlations between magnitude of the suppression effect and movement speed at the time of stimulation. Data is plotted separately for both groups receiving a strong tactile feedback **(left side panels)** and a weak tactile feedback **(right side panels)**, as well as visual and tactile feedback. With strong tactile feedback, both the visual- and tactile-late condition (darker lines on the left panels) showed a significant correlation.

Finally, Figure 6 shows within-condition correlations between movement speed at stimulation and suppression scores. In the group receiving strong tactile feedback, we found a positive relationship between speed at time of stimulation and suppression in the late phase of the movement both for visual, *r* = 0.45, *p* = 0.023, and tactile feedback, *r* = 0.40, *p* = 0.043.

## Discussion

In the present study, we aimed to investigate whether tactile suppression is modulated by (a) the task-relevancy of predicted somatosensory signals and (b) the intensity of these signals. We asked participants to reach to an invisible target in a cued area in the air. We varied the task-relevancy of predicted somatosensory feedback by providing either a visual or a tactile signal to indicate that the reach was successful. In addition, we varied the relative difficulty of detecting the tactile feedback by varying its intensity: one half of the participants received strong tactile feedback, while the other half received weak tactile feedback. Tactile probing stimuli were applied to the reaching finger, either early or late in the movement. We found that tactile suppression decreased in the late compared to the early phase of the movement. With strong, tactile feedback, suppression was more pronounced compared to visual feedback. There was no difference between weak tactile and visual feedback. Overall, our results support the view that tactile sensitivity is upregulated when the predicted somatosensory consequences of a movement are task-relevant *and* associated feedback signals are faint. Such modulation of tactile sensitivity is advantageous, as it allows one to detect stimuli that would otherwise be missed. We suggest that such modulation may be characterized as a “release” from suppression if it is potentially resource-intensive to implement, and thus only invoked when such expenditure is required for successful goal-directed actions.

A novel component of this study was our attempt to reduce predictable touch at the end of a goal-directed movement. Predicted action effects can result in an increase in tactile suppression in the late phase of a movement, when manual contact becomes more likely (Juravle et al., 2010; Fraser and Fiehler, 2018; Voudouris and Fiehler, 2021). This could be explained by a forward model which suppresses somatosensory feedback when it can be reliably predicted, based on future sensory states of the moving body part to free capacities for efficient information processing (Desmurget and Grafton, 2000; Williams and Chapman, 2002; Bays et al., 2006; Chapman and Beauchamp, 2006). Certainly, such movements are characterized by highly detectable tactile outcomes. However, the increase in late reach suppression reported in these studies could also be attributed to a lack of task-relevance of the tactile feedback. For example, in the study by Fraser and Fiehler (2018), participants suppressed *more* when given paired tactile and visual feedback about task completion, compared to visual only feedback. In this case, the additional tactile feedback did not provide unique task-relevant information, as participants could solely rely on visual feedback. In contrast, reduced suppression can be found in haptic exploration when tactile information must be actively obtained for task completion (Juravle et al., 2013). Overall, it appears that increased suppression at the end of a movement is primarily related to the expectation of touch at the end of the reach that is not uniquely relevant to task performance.

Other studies have reported decreased tactile suppression at the end of a goal-directed movement (Colino and Binsted, 2016; Colino et al., 2017; Juravle et al., 2018). For example, during grasping the strength of tactile suppression is locally dependent and, thus, less pronounced on the index finger than on the pinky or the forearm of the involved limb (Colino et al., 2014; Colino and Binsted, 2016; Juravle et al., 2018). These data also suggest that suppression on the moving hand seems to be reduced when feedback signals become more relevant for the ongoing task (Colino and Binsted, 2016; Manzone et al., 2018; Voudouris and Fiehler, 2021). Theories of motor control, such as optimal feedback control, assume that sensory feedback is modulated according to the goals of an ongoing action (Todorov and Jordan, 2002; Liu and Todorov, 2007). An optimal estimate of the system’s state is predicted by combining sensory feedback and estimates from a forward model. Optimization of motor output is than accomplished by a controller that tunes feedback gains. Both predictive mechanisms and somatosensory feedback processing are combined in goal-directed movements to enable optimal action planning and flexible responses to incoming sensory information (Voudouris and Fiehler, 2021). A reduction in suppression could be explained by an increased reliance on somatosensory feedback when it is necessary to perform the task. Depending on the task, this may affect different timepoints in the course of the movement and thus explain the temporal differences of tactile suppression found in different studies. Altogether, the relevance of processing somatosensory signals to successfully accomplish a task appears to be important in modulating tactile suppression, beyond the prediction of tactile stimuli in general.

In the present study, we observed stronger suppression in the early, compared to the late phase of the reach, although a predictable action effect was present only at the end of the reach. However, this action effect was much weaker compared to previous studies (Fraser and Fiehler, 2018), which included contact with a surface at the end of the movement, creating a highly predictable and detectable tactile consequence that may have increased late-reach suppression. Excluding this predictable touch signal at the end of the movement significantly reduced the temporal suppression profile. As the movement had to be stopped before the screen, participants needed to wait for visual or tactile confirmation that their movement was successful. Thus, the received feedback was uniquely relevant for the task and participants were required to detect it. For tactile feedback in particular, tactile suppression would impede task performance. In line with previous studies (Juravle et al., 2010; Voudouris et al., 2019), in our task participants had to rely on feedback signals to successfully accomplish their goal. This feedback processing is increasingly necessary in the late phase of the reach in order to appropriately control the movement and detect task-relevant feedback signals. This may explain a reduction in suppression in the late phase of the movement, compared to the early phase. To the best of our knowledge, our study was the first to show this pattern of suppression without a predictable touch at the end of a movement. Thus, our results suggest that it is not the predictable action effect alone being suppressed, but that the need to process task-relevant feedback signals that would otherwise be missed influences the strength of tactile suppression.

Further, the modality of received feedback influenced tactile suppression. As expected, we observed an overall increase in suppression for tactile compared to visual feedback, both in the early and in the late phase of the movement. This differential effect argues against a general gating mechanism, leading to an overall suppression of external somatosensory signals during movement (Press et al., 2020; Kilteni and Ehrsson, 2021). The increase in suppression for tactile compared to visual feedback points to a predictive component based on the expected tactile feedback at the end of the reach. Together with other studies (Fraser and Fiehler, 2018; Gertz et al., 2018; Manzone et al., 2018; Führer et al., 2021; Voudouris and Fiehler, 2021), our results support the assumption that suppression of external tactile probing stimuli cannot be explained by unspecific gating alone but is adjusted to predicted somatosensory consequences of the movement.

This assumption is further supported by the dependency of suppression on the intensity of tactile feedback, as we only observed a difference between visual and tactile feedback when tactile feedback was strong and not when it was weak. Suppressing feedback signals that are already weak and thus harder to detect would increase the chance of missing relevant somatosensory information. As a consequence, weak feedback signals become less reliable, which has been associated with weaker suppression (Blakemore et al., 2000; Klever et al., 2019; Voudouris et al., 2019). Overall, it seems conceivable that feedback signals need to be stronger than the sensory noise associated with processing of afferent signals to be detected (Blakemore et al., 2000; Voss et al., 2008; Voudouris et al., 2019). If tactile suppression is strong, weak tactile consequences of movement will go undetected. In cases where such consequences are valuable to the moving agent (i.e., they are task-relevant), downregulating the predictive mechanisms which give rise to tactile suppression can improve task performance. This downregulation is only useful if the agent receives a marked improvement in detection. If somatosensory input signals are already strong, further increasing their strength does not seem to increase tactile suppression (Broda et al., 2020). Further research is needed to investigate whether there is a specific sensory threshold for somatosensory feedback signals to be suppressed, or whether there is a continuous relationship between downregulation and the apparent benefit of improved sensitivity.

Reach characteristics did not depend on the intensity of the tactile feedback received at the end of the movement; reaches were comparable between the two feedback groups. Movement speed profiles show that participants tended to decelerate fairly early in the movement on trials with a probing stimulus occurring in the early phase of the movement, compared to the late phase. This was also reflected in longer reaching times for the early stimulation condition. We speculate that this deceleration may be an effect of the probing stimulus, that is, participants are slowing down as they register or react to the sensation. The difference in reach time between the early and late stimulation condition was even more pronounced with visual feedback, compared to tactile feedback, pointing out a stronger deceleration in movement speed for this condition. As there was no strong expectation of a tactile sensation in the visual condition, the early probing stimulus may have been more surprising and therefore resulted in more online adjustment of the movement. These results raise an interesting question about how reach characteristics may be influenced by the detection task itself, which inherently distracts from the movement. However, as stronger suppression occurred consistently at both stimulation times for tactile feedback, movement differences alone cannot explain the differences in tactile suppression found for the two feedback types.

In addition to these differences in movement characteristics, the highest suppression occurred in the early phase of the movement together with the time of maximum speed during the reach. Interestingly, there was no correlation between movement speed and suppression in the early, but in the late phase of the movement. Only when tactile feedback was strong we observed a positive relation between speed at stimulation and suppression. At the same time, speed at stimulation time was greatly reduced in the late phase of the movement, even more in anticipation of tactile compared to visual feedback. Overall, higher speed can therefore not explain the increase of suppression during movement. The previously reported positive relationship between the speed of movement and the amount of tactile suppression appears rather context-dependent (Angel and Malenka, 1982; Schmidt et al., 1990; Cybulska-Klosowicz et al., 2011). Since there was no contact with a surface in our current experiment, an increase in the expected force due to higher speeds (e.g., by hitting the surface harder) cannot explain the correlation with suppression scores in the late phase of the reach. In addition, the relationship between suppression and speed at time of stimulation occurred equally for both feedback modalities and yet there was more overall suppression in the tactile condition. This again argues against the assumption that differences in movement trajectories alone can explain the differences in suppression. Rather, the partly contrasting results of different studies regarding the extent of suppression, especially at the end of a movement, might reflect different demands in somatosensory processing caused by specific movement conditions.

## Conclusion

In summary, the present study supports the view that tactile suppression is based on a predictive mechanism, which is modulated by the need to process incoming somatosensory information that is both task-relevant and difficult to detect. Consequently, tactile suppression can to some extent be adapted to different demands in somatosensory processing. We showed that a reduction in predictability of an action effect at the end of the reach can lead to a general reduction in tactile suppression. Nevertheless, the mechanism proved sensitive to the modality of received feedback. Tactile suppression was more pronounced with tactile feedback, compared to visual feedback. Further, a reduction in tactile suppression was shown by reducing the intensity of anticipated feedback signals, suggesting that downregulation occurs when the consequences of missing a weak movement sequence are severe. We conclude that it is not the predicted action effect alone which influences the extent of tactile suppression, but rather the need to detect and process predicted feedback signals occurring during the movement which matters most.

## Data Availability Statement

The raw data supporting the conclusions of this article are publicly available at https://doi.org/10.17605/osf.io/v7q9n.

## Ethics Statement

The studies involving human participants were reviewed and approved by Research Ethics Board, Department of Psychology and Sports Science, Justus Liebig University Giessen. The participants provided their written informed consent to participate in this study.

## Author Contributions

MB: data curation, formal analysis, investigation, project administration, software, validation, visualization, roles and writing—original draft, and writing—review and editing. LF: conceptualization, data curation, methodology, project administration, software, and writing—review and editing. KF: conceptualization, funding acquisition, methodology, project administration, resources, supervision, and writing—review and editing. All authors contributed to the article and approved the submitted version.

## Conflict of Interest

The authors declare that the research was conducted in the absence of any commercial or financial relationships that could be construed as a potential conflict of interest.

## Publisher’s Note

All claims expressed in this article are solely those of the authors and do not necessarily represent those of their affiliated organizations, or those of the publisher, the editors and the reviewers. Any product that may be evaluated in this article, or claim that may be made by its manufacturer, is not guaranteed or endorsed by the publisher.
